# Neuro-Inspired Spike-Based Motion: From Dynamic Vision Sensor to Robot Motor Open-Loop Control through Spike-VITE

**DOI:** 10.3390/s131115805

**Published:** 2013-11-20

**Authors:** Fernando Perez-Peña, Arturo Morgado-Estevez, Alejandro Linares-Barranco, Angel Jimenez-Fernandez, Francisco Gomez-Rodriguez, Gabriel Jimenez-Moreno, Juan Lopez-Coronado

**Affiliations:** 1 Computer Architecture and Technology Area, Universidad de Cádiz, School of Engineering, Calle Chile, 1, Cadiz 11002, Spain; E-Mail: arturo.morgado@uca.es; 2 Robotic and Technology of Computers Lab (RTC), Universidad de Sevilla, ETSI Informática, Avd. Reina Mercedes s/n, Sevilla 41012, Spain; E-Mails: alinares@atc.us.es (A.L.-B.); ajimenez@atc.us.es (A.J.-F.); gomezroz@us.es (F.G.-R.); gaji@us.es (G.J.-M.); 3 Automation and System Engineering Department, Polytechnic University of Cartagena, Campus Muralla del Mar, Cartagena, 30202, Spain; E-Mail: jl.coronado@upct.es

**Keywords:** spike systems, motor control, VITE, address event representation, neuro-inspired, neuromorphic engineering, anthropomorphic robots

## Abstract

In this paper we present a complete spike-based architecture: from a Dynamic Vision Sensor (retina) to a stereo head robotic platform. The aim of this research is to reproduce intended movements performed by humans taking into account as many features as possible from the biological point of view. This paper fills the gap between current spike silicon sensors and robotic actuators by applying a spike processing strategy to the data flows in real time. The architecture is divided into layers: the retina, visual information processing, the trajectory generator layer which uses a neuroinspired algorithm (SVITE) that can be replicated into as many times as DoF the robot has; and finally the actuation layer to supply the spikes to the robot (using PFM). All the layers do their tasks in a spike-processing mode, and they communicate each other through the neuro-inspired AER protocol. The open-loop controller is implemented on FPGA using AER interfaces developed by RTC Lab. Experimental results reveal the viability of this spike-based controller. Two main advantages are: low hardware resources (2% of a Xilinx Spartan 6) and power requirements (3.4 W) to control a robot with a high number of DoF (up to 100 for a Xilinx Spartan 6). It also evidences the suitable use of AER as a communication protocol between processing and actuation.

## 1. Introduction

Human beings, and their ancestors before them, have evolved throughout millions of years and obviously their systems to perform tasks too. Most of these tasks are commanded by the brain. Therefore, engineers, and specially the neuromorphic engineering community [1,2] have fixed as their main goal to mimic the human systems which are supposed to have an extraordinary behavior carrying out their own tasks.

In particular, reaching movements (planning and execution) have been for ages one of the most important and studied ones [3]. If we take a closer look in humans, we will find that the system involved in these tasks is the central nervous system (CNS). This system is a combination of the brain and the spinal cord and, simplifying, it consists of neuron cells and uses spikes or graduated potentials to transmit on the information across the anatomy [3].

Nowadays, it is possible to integrate several thousands of artificial neurons into the same electronic device (very-large-scale integration (VLSI) chip [4], Field-Programmable Gate Array (FPGA) [5] or Field-Programmable Analog Array (FPAA) [6]); which are called neuromorphic devices. There are many European projects focused on building computing systems which exploit the capabilities of these devices (Brain-inspired multiscale computation in neuromorphic hybrid systems (BrainScale; website: http://brainscales.kip.uni-heidelberg.de/index.html), SpiNNaker (website: http://apt.cs.man.ac.uk/projects/SpiNNaker/) and the Human Brain Project (HBP; website: https://www.humanbrainproject.eu/) as examples). One of the main challenges is which devices and how to integrate them to produce functional elements.

One of the problems faced when we try to integrate and implement these neural architectures is the communication between them: it is not easy to distinguish which neuron of what device is firing a spike. To solve this problem, new communication strategies have been exploited, such as the Address-Event- Representation (AER) protocol [7]. AER maps each neuron with a fixed address which is transmitted through the interconnected neuronal architecture. By using the AER protocol, all neurons of a layer are continuously sharing their excitation with the other layers through bus connections; this information can be processed in real time by a higher layer.

AER was proposed to achieve communication between neuromorphic devices. It tries to mimic the structure and information coding of the brain. Like the brain, AER will let us process information in real time, by implementing simple spike-based operation at the time each spike is produced or received. That's one of the reasons for using it: the intrinsic speed behind the spike-based philosophy. Another one is the scalability allowed by its parallel connections.

The motion problem is still being widely studied. One successful approach to these control architectures, including visual feedback, dealing with motion problem was based on “visual servoing” where a camera guides the arm movement computing complex algorithms [8,9]. Nowadays, this system is still used in industry due to its reliability, but these systems were based on high resource consumption computational models instead of low resource neuroinspired spikes-based models. In this work, we describe the steps taken towards a fully neuroinspired architecture. Once the first approaches to bio- inspired image sensors appeared a few years ago (early 2000s) [10], and the race to make a complete system began. Until those days, there were some advances in describing neuroinpired control algorithms: in [11] a couple of them were shown: one to generate non-planned trajectories (Vector Integration To-End Point—VITE) and the other one to follow them by muscles (Factorization of LEngth and TEnsion—FLETE). Then, many related works using them were published [12–14]. All of these works listed were based on simulations, but this work presents a real spike-based hardware implementation of VITE.

Recent works show a hardware implementation of these algorithms: In [15] they use the same framework (algorithm and platform) as this article and they deal with the problem of two frames of reference, one for visual and the other one for the robot by doing a mapping between them. Then, in [16] a whole pseudoneural architecture is designed and applied to an iCub robot [17]; one of the algorithms selected was VITE [18]. In [19], they include the joint limits using the Lagrange theorem. The great and simple control achieved at least shows the opportunities of using the VITE algorithm. Two of the nearest works to this paper from close research groups are in [20], where they used both algorithms but with a PC to run the equations and find the best way, and also in [21], where a spike processing Proportional Integral and Derivative (PID) control is implemented; it is in this last article where the Pulse Frequency Modulation (PFM) modulation for motor running appears.

Most of the listed works used a computer to process separately at least one element of the dynamic system. Also, the computing mode was not spike-based. This provokes delays, non-real time [22] and definitely it is not an entirely bioinspired behavior, although that was the original thought.

This article is focused on real time planning, execution and motion control in a bioinspired way: to design a fully neuroinspired architecture from the retina to the robot. We have set two constraints: only spikes can flow across the system and only addition, subtraction and injection of spikes are allowed. In this way, copying these neural constraints, we achieved a neuro-inspired control. We support the spike processing method for all the algorithms used; that is the main claim of this paper: design, development and implementation of a spike-based processing control architecture and to avoid using an external computer for processing, with extremely low power consumption and AER communication.

First of all we have to determine the elements to integrate according with the biological principles: image sensor, a hardware architecture where the CNS behavior is emulated and a robot to execute the movements. There are many sorts of problems to deal with in this selection: sensor must be a spiking retina, the architecture has to keep as many CNS features as possible, within only addition, subtraction and injection of spikes, and finally, the robotic platform will be made of motors which mimic the muscles.

The first element of the architecture is the image sensor. We have chosen a silicon retina, the dynamic vision sensor developed by the Tobi Delbruck research group [23]. It is a VLSI chip made of 128 × 128 analog pixel firing spikes (with AER protocol) when a threshold is reached.

Applying several processing layers to these events flow [24], a single event, which meets the center of an object, is isolated. Therefore, this event plays the role of the target position for the system, so the retina will deliver the reaching position to the architecture.

The main part of the system turns around the VITE algorithm [11]. It was selected because it is inspired by the biological movement and was designed to mimic it. It has been translated into the spikes domain using spike-based building blocks which add, subtract or inject spikes like the human neural system. This algorithm generates a non-planned trajectory and it needs a second algorithm to produce and control the forces applied to the motors which mimic the muscles. In this paper we are focused on the first algorithm and therefore, no feedback is performed. Our aim is to evaluate the viability of translating the VITE completely into the spikes domain and applying it to a real robotic platform in order to enable the second algorithm and to close the control loop.

In order to achieve the described goal, we have transformed the algorithm using existing spike processing blocks developed for our research group [21,25,26] and put them into MATLAB Simulink to test and adjust the blocks. Afterwards, two FPGA based boards were used to allocate the blocks and mimic the biological structure (one for the brain and the other one for the spinal cord). The final architecture results in the retina connected to the processing FPGA board, this one to the actuation FPGA board and finally to a stereohead robotic platform.

The main achieved result is that it is possible to control the robotic platform in an open loop way by mapping the information received by the retina with the expected movement at the robot. There is high accuracy between the simulated curves and the signals read from the motors encoder.

Finally, avoiding the use of the second algorithm causes a new component implemented in the second FPGA board. The task of this component was to adapt the spikes into a Pulse Frequency Modulation (PFM) modulation to feed the motors.

The rest of the paper is structured as follows. Section 2 presents the research inspiration and motivation: how an intended movement is produced from a biological point of view. Then, the background for VITE algorithm is described and the spike-based processing is presented. The next section translates the VITE algorithm into the spike-based paradigm, the SVITE: spike-based VITE. Then, the implementation of the SVITE into the FPGA boards dealing with the hardware advantages and disadvantages is presented. In the next section, the results performed are shown and the accuracy of translation is discussed based on the results obtained. The last two sections are devoted to the discussion, which includes the connection between the electronic algorithm and the biological movement, and future directions for this research.

## 2. Biological Movement

### 2.1. Introduction

In this section we are going to present a short review of how an intended movement is performed by humans. Just the first stage of the motion will be described: the one connected with the brain; we leave the passive and reflex movements aside because they are not executed from a sighted target.

Our starting-point is the human central nervous system (CNS) that plays the role of movement controller. The CNS consists of the brain and the spinal cord [27]:

- The brain integrates the information from the spinal cord and motor cortex in order to plan, coordinate and execute the desired movements. This article covers the function developed by this part.
- The spinal cord receives information from several sensory elements and includes the motor neurons in charge of intended and reflected movements and the tracts for the information flow.

Focusing in the brain, inside the cerebral cortex, there are two systems, with three areas per each, responsible for processing the sensory and motor information: sensor and motor system. The areas are called primary, secondary and tertiary depending on the abstraction level they manage.

The motor system has to process the information about the external world presented by the sensory system and project it into the neural elements to carry out the movement.

**Figure 1.**
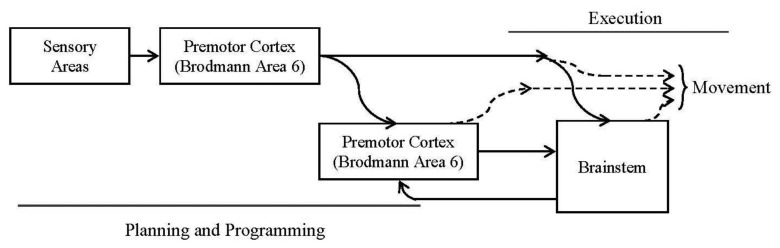
Simplified block diagram for intended movements' execution. There are two parts: one for the planning and programming (involved in this article) and the second part for the movement execution. In the diagram, the arrows represent the information flows: solid lines are used to represent trajectory information and dotted lines are used to represent movement commands. In this article, the second part has been implemented as a wire; neither feedback has been considered.

To carry out these tasks, motor systems have a continuous sensory information flow and a hierarchical organization of three levels: the spinal cord, the brainstem and the motor cortex areas [27]. This organization is also massively connected and has feedback at all of its levels. A brief description of these areas is as follows:

- The spinal cord is the lowest level in the hierarchy. It has neural circuits to produce a wide range of motor patterns. The motion in this level can occur even when it is disconnected from the brain [3].
- The brainstem is responsible of driving the neural systems. All connections between the spinal cord and the brain go through the brainstem and across two parallel tubes.
- Related to the motor cortex, we shall consider these three subareas: primary motor cortex (Broadmann area 4), premotor cortex (part of Broadmann area 6) and supplementary motor cortex (actually it is part of the premotor cortex). This last area projects directly to the spinal cord. The rest of the areas project to the spinal cord through the brainstem. Figure 1 represents the described behavior.

### 2.2. Intended Movements

The upper elements of the motor system (motor cortex) are responsible for: motion planning according to target and environment information. To do so, there are many projections between sensory areas and motor cortex (primary and premotor). The sensory areas may integrate information from different kinds of sensors and project directly to the premotor cortex. It will receive information of the reaching target and it has two different areas:

- Supplementary motor area: It plays an important role in complex movement executions and in movement practices.
- Premotor cortex: It controls the reaching movements and it can project to the primary motor cortex and directly to lower motor controller instances.

In the sixties, an activity in the primary motor cortex before the movement execution was observed. It was due to a planning of the movement in progress [28]. There is one more subcortical structure type playing an important role in motion control: the basal ganglia [29]. They receive inputs from the neocortex and project to the brainstem controlling the movement in progress. Although they play a role, it is still largely unknown and we have not introduced it in our model.

Figure 1 shows a block diagram for the intended movements' production:

## 3. Vector Integration to Endpoint (VITE) Algorithm

### 3.1. Introduction

Any simple action involves the use or coordination of hundreds of biological elements. A joint movement for example, will cause coordination between two or more muscles. At the same time, each muscle consists of several fibers and sensory cells connected to efferent and afferent nerves coming down from the spinal cord. The VITE algorithm [11] tries to model the human movements keeping as many details of the neural system in mind as possible.

Essentially, VITE generates the trajectory to be followed by the joint, but in contrast to approaches which require the stipulation of the desired individual joint positions, the trajectory generator operates with desired coordinates of the end vector and generates the individual joint driving functions in real-time employing geometric constraints which characterize the manipulator.

Notice that VITE is the first layer involved in a planned arm movement. It does not integrate any feedback from the end robot. It generates the trajectory regardless of the forces needed to develop the movement. Thus, it feeds a theoretical second layer commanded by another algorithm [12]. This second layer contacts with the end manipulator and manages the command received by the previous one.

### 3.2. Block Diagram and Equations

**Figure 2.**
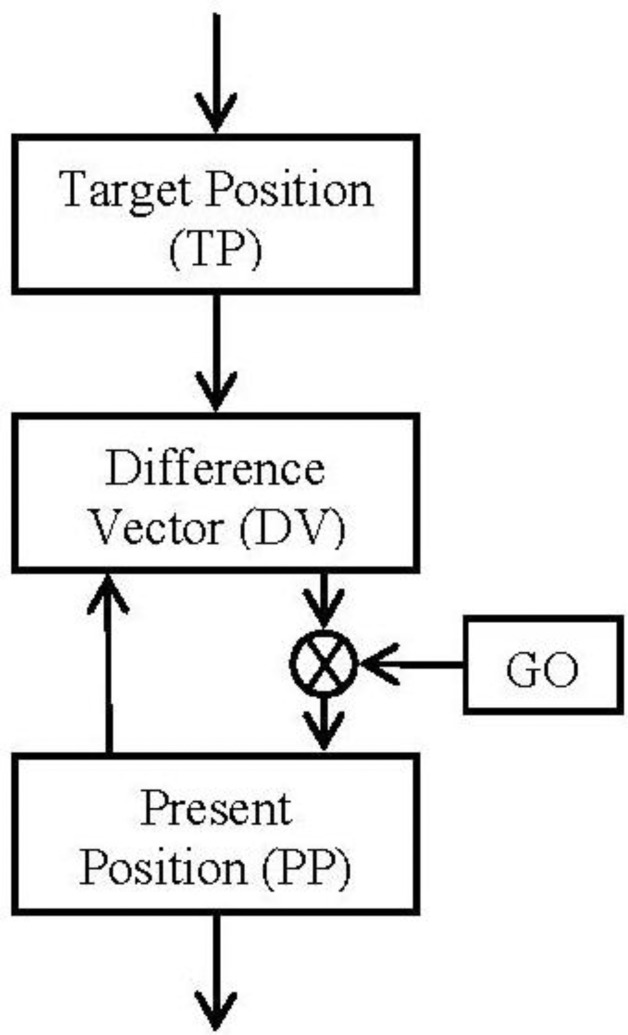
Block diagram. Taken from [11].

The block diagram (Figure 2) and the equations are presented in this subsection in their simplest form for the algorithm. The algorithm will integrate the difference vector at each time in order to update the present position (Equation (2)). But it will not be updated until the GO signal has a non-null value (Equation (2)). Meanwhile (GO signal has a null value) the difference vector is pre-computed in order to be ready for the shoot in the control signal. This time is known as the “motor priming”. If at any time while the movement is being done the GO signal goes zero, the movement will be frozen in that position.

The target position can be updated during the movement. This change will cause just an update in the difference vector regarding the new goal.

All the positions pointed in the algorithm must be referred to the same frame. Therefore, if spatial positions are considered, the integration of the present position (PP) will be matched with the speed profile of the movement.

If we make a comparative between this algorithm and the classical control theories for industrial applications (Proportional, Integral and Derivative controllers), this algorithm would result similar to classical integral controller due to the final integral component but it is not. This integral plays the role of the end robot to feedback the ideal position reached. The special component, GO signal, carries out a pseudo proportional playing the role of a pseudo disturbance.

### 3.3. Some Considerations for the Algorithm Application

#### 3.3.1. Synchronous Movement

The algorithm faces the preconceived theories that talk about a preprogrammed trajectory before a movement is done. With this algorithm, the movements are carried out in a real time and it is possible to change the target during the movement without disturbing it. Also, in the introduction we stated that this algorithm is intended to cover a complete movement involving several muscles; a joint for example.

**Figure 3.**
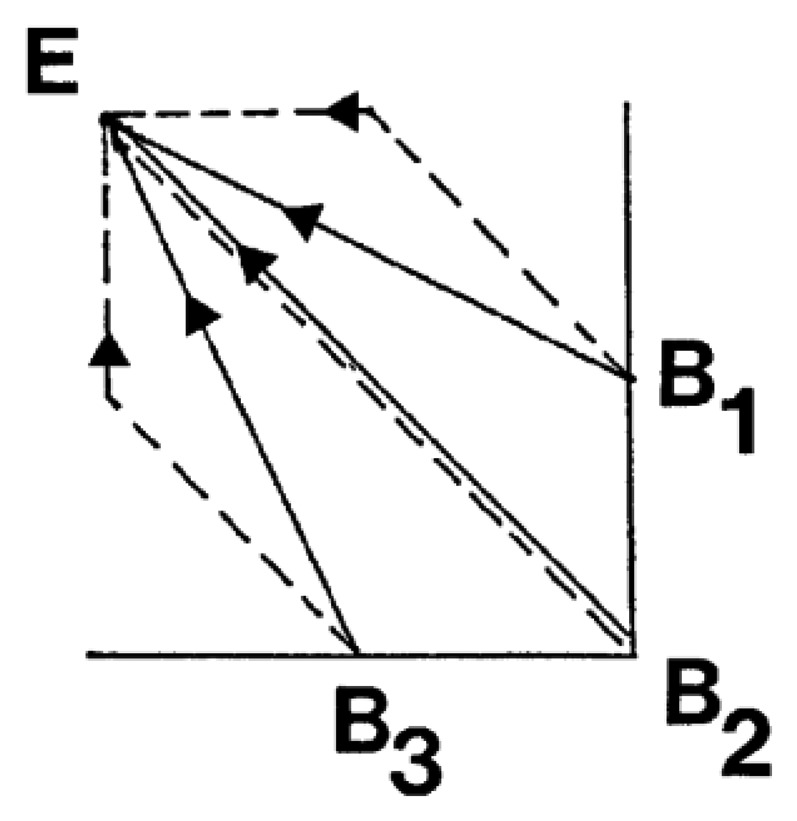
Three movements composed of two joints are represented. The start points are B1, B2 and B3 and the end point is the same for all of them: E. Solid lines represent the right way to perform the movement and the dotted lines indicate a composite of two actions. This is a graph taken from [11].

Thus, it receives an abstract reference, *i.e.*, a spatial point and it should generate a trajectory for all the muscles involved in the action. In addition to this, the movement cannot be a composite of two or more joint movements (dotted lines in Figure 3); it must be a gesture or synchronous action of various muscles (solid lines in Figure 3). This concept is called synergies and they happen in a natural and dynamic way.

Thus, to perform a synchronous movement, each muscle group should contract or expand at a different quantity according to the difference vector computed for each one. From this concept of synchronous movement comes up the need for pattern and speed factorization. With an independent speed control for each muscle group it is possible to adjust all of them to reach high accuracy in the synchronization between all the muscles involved in the movement.

### 3.3.2. Coding the Position

One important issue regarding this algorithm is how to code the position of an isolated muscle or a whole joint. From [11], two methods can be used to know the position of the end muscle: the corollary discharge and the inflow information. The first one is the command provided to the muscle and from the brain. The second is the feedback information from the muscle.

Therefore, with the corollary discharges it is supposed that the end effector arrives to the ordered position and with the inflow information it is possible to update the position if a passive movement is performed and also to check the position in an intended movement.

The VITE algorithm uses only the signal from the brain to update the present position and therefore, to generate the trajectory. Thus, it is supposed that the end effector reaches the commanded position. This is a typical way of a repetitive movement. However, regardless of whether the inflow information exists or not, it is necessary to implement gates to inhibit or allow it.

#### 3.3.3. GO Signal and Speed Profile

The GO signal is in charge of the movement speed control. It is also the gate for that movement. The implementation of this signal causes a different speed profile in the global movement. The typical signal used is a ramp; the higher the slope, the faster the movement.

With a ramp profile, the general speed profile achieved is a bell-shaped one. At the beginning speed is low. When the target is being reached the speed increases. At the end of the movement the speed goes down to increase the accuracy. The symmetry of this bell shaped profiles vary with speed [30]. Notice that this signal loses its meaning when the target is reached. To sum up, it can be said that it is not important how to reach a target, but just to reach it. So, the trajectory does not matter, except for fitting the joint angle constraints.

## 4. SVITE: Spike-Based VITE

### 4.1. Spike-Based Processing

This section presents a brief description of spike-based processing. This way of processing aims to mimic the behavior of the human nervous system. The information in this system is analogue and we try to reproduce it, but with digital devices. The design is made up of a hardware description language (HDL) of several blocks. These blocks process the information in the simplest way: addition, subtraction and injection of spikes are allowed as this is supposed to be in a biological neuronal process. The information is based on the firing rate of the blocks trying to mimic the human neurons analogue. There are only spikes flowing between these blocks, being processed while they flow, until they are applied to the motors.

This processing way takes advantage of the higher clock frequency of these digital systems to achieve an equivalent processing. The principal advantage is its simplicity; we do not need complex processors to solve equations. Also the power consumption is an important point: there is a huge difference of watts between the typical process computer and the electronic elements. Another profitable advantage is space: we use small electronic devices which could be allocated in a stand-alone way. There is a published work [21] which reveals the power of this spike-based processing, where a spike PID controller was designed and tested.

### 4.2. Translation into the Spikes Paradigm

In a previous section, the VITE algorithm has been presented and thoroughly described. This section presents the translation of the algorithm into the spikes paradigm. We have called this new algorithm SVITE, for spike-based VITE. The translation is done in two ways: keeping the information in a spike-based system in mind and taking advantage of the Laplace transformation to solve the equations considering zero initial conditions.

In these spike systems, the information has a relation with the inter spike interval (ISI) and specifically with the firing rate which can be understood as the frequency. That is the reason why we first go into frequency domain with Laplace (we are not matching Laplace domain with firing rate, it is just an interpretation to let us translate into spike-based processing paradigm).

**Figure 4.**
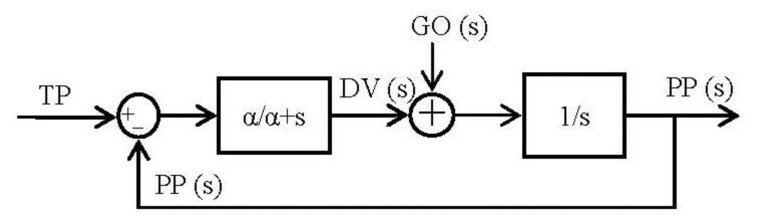
Block diagram resulted from the conversion of the VITE algorithm using the Laplace transformation. The low pass filter (with single gain) block avoids abrupt changes in the error signal and the integer block performs the robotic platform tasks. This block simulates the robotic platform and therefore it makes possible non-feedback from the robot. The robot is supposed to reach the commanded position.

Therefore, taking the equations of the algorithm as our starting-point and using the Laplace transformation to solve the equations, the main parts of the algorithm are translated regardless of whether the GO signal is used, because if we translate the equations in a strict way, the product between GO signal and the difference vector will be translated into a convolution in the frequency-domain and it will not be correct because this GO signal was designed in order to control the speed of the movement in the original algorithm. With this argument and regarding the information inside a spike system, the translation into spike paradigm for this product will be an addition of two spike trains in the spikes-processing paradigm. As a result, the firing rate (or frequency) of the resulting signal will be increased in any case. The next section deals with this idea. Thus, the translation, starting with Equations (1) and (2), is as follows:

By running Equations (5) and (6) it is easy to arrange them in blocks (Figure 4):

(1) $$\frac{d}{dt}DV=\alpha \times \left(-DV+TP-PP\right)$$

(2) $$\frac{d}{dt}\;PP=DV\;x\;GO$$

(3) s×DV(s)=α×(−DV(s)+TP(s)−PP(s))

(4) s×PP(s)=DV(s)

(5) $$\text{DV}\left(s\right)=\frac{\alpha }{1+\alpha }\times \left(TP\left(s\right)-PP\left(s\right)\right)$$

(6) $$\text{PP}(s)=\frac{1}{s}\times DV\left(s\right)$$

**Figure 5.**
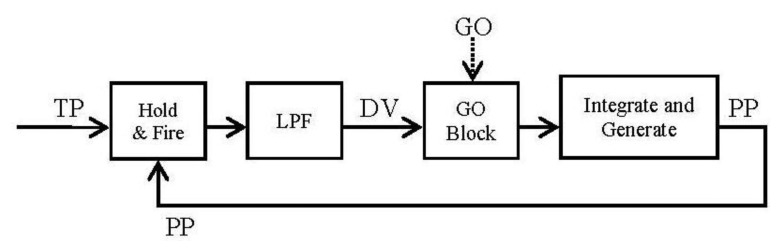
Block diagram generated from existing spikes processing blocks. The Spike Hold & Fire block performed the subtraction between the present position and the target position; both signals are spike streams. The GO block allows speed control of the movement and it will be explained in the next section. The Spikes Integrate & Generate (I & G) block allows us to integrate the DV (Difference Vector) signal (again a spike stream). This block is composed by a spike counter and a spike generator. The latter uses a parameter called Integrate & Generate Frequency Divider (IG_FD) to divide the clock signal and generate the output stream according to this division.

Once we have the block diagram of the algorithm in the frequency domain, going through [26,31] the resulting spike blocks are detailed in Figure 5.

The GO block is the most special one because it has to deal with the problem of trying to mimic a biological behavior. It is not taken directly from the Laplace transformation due to its exposed particularity. It is thoroughly described in the next section.

### 4.3. Go Block

The main function of this block is to control the speed of the movement and also to be the gate of it, but it has to deal with the fact that thinking in neuromorphic engineering it is not allowed to carry out a multiplication as usual because it is not a biological behavior.

In the spike-based information codification, an approach to perform the GO function is to inject a determined number of spikes every time the previous block fires one, but equidistantly distributed over time as much as possible. It is like amplifying or increasing the activity, thinking in a biological way.

**Figure 6.**
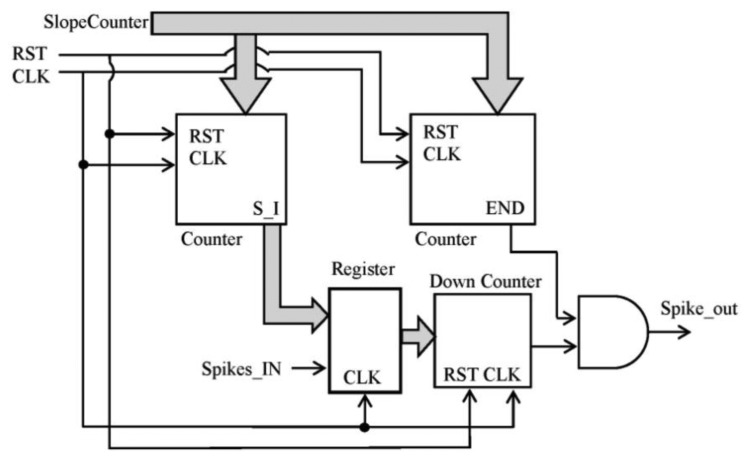
Block diagram generated for the block. It includes three counters: two of them are straight: one for the number of spikes to inject (S_I in the diagram) and another one for the life signal (it will produce the END signal to finish the movement); the last one is a decreasing counter in order to inject the accurate number of spikes.

There are a few options to implement the block into the FPGA: to inject spikes according to the slope of a ramp, just one to N spikes per each received in a continuous way and so on. It can be implemented following any other function, but we have selected the ramp because it allows speed control and it is quite simple to implement inside the FPGA with counters. This selection let us configure the slope to achieve the desired speed. The final synthesized block is described in Figure 6.

**Figure 7.**
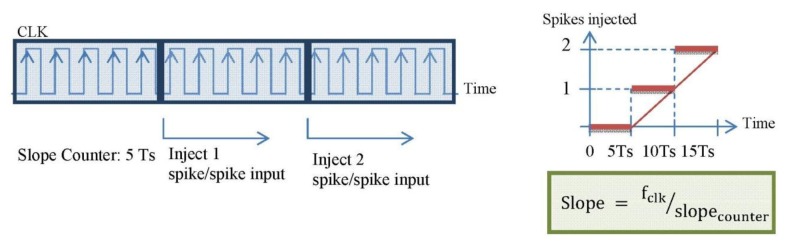
Explanation diagram of the implemented block. In this example, the slope counter is fixed to five clock periods; every time the count is reached one more spike will be injected. This way, and considering the firing rate, the discrete solid line is performed, and we were looking for the thinner line behavior.

The straight counters receive the slope_counter parameter and produce the number of spikes according to the slope value of the ramp and the signal to finish the spike injection, respectively. Every time that a spike is received, the register value is updated with the number of spikes to inject. Figure 7 shows the behavior explained.

**Figure 8.**
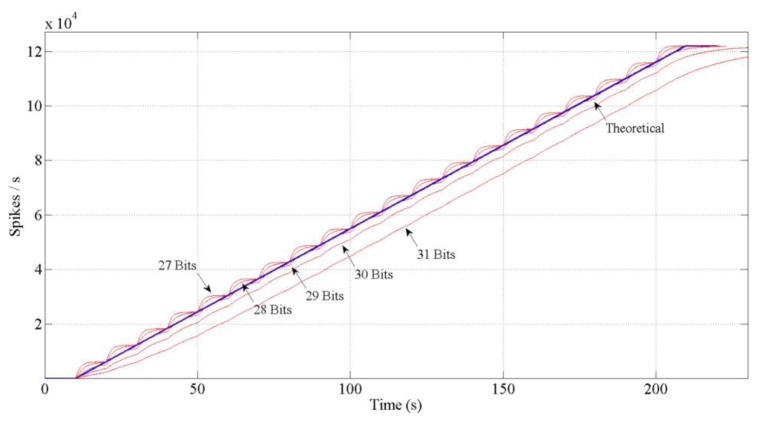
Comparison between five different bits implementations of the integrate and generate in the low pass filter. The theoretical behavior is also represented. The results performed with 27, 28 and 29 bits for the counter show a jumping behavior due to the saturation of the integrator. The higher the number of bits used, the slower the behavior. There is a trade-off between the desired speed and the avoided saturations. The slope used was 0.1% and the input 6.1 Kevents/s. In this case, the number of bits selected would be 29 (10% slope will use 21 bits).

If we design the block as it has been explained, the red thicker line behavior in Figure 7 would be performed. It is a discrete result. A logical conclusion if we consider the spike systems: to inject or not a spike. Then, to reach the continuous solution (thinner line) it is necessary to include a low pass filter (spike-based and with single gain, too) at the output in the block diagram. This filter will distribute the spikes uniformly. However, including this filter involves a problem: the spikes low pass filter includes an “integrate and generate” (also spike-based) [31] at its output. This I & G block keeps an n-bits count for the incoming spikes and generates spikes according to that count. So, we have to avoid the overflow of the count. Thus, we should finely tune it in order to avoid the saturation of the whole system. Figure 8 shows different bits implementations for the integer.

In any case, latency at the beginning will be calculated from the first count that injects any spike. During this period, the Difference Vector (DV) will be calculated by the previous part of the algorithm. Also, this time it is consistent with the fact that in a biological movement a previous activity is detected in the premotor cortex [28]. The latency is defined as follows:

(7) $$\text{latency}=\frac{\text{slope}\_\text{counter}}{f_{\text{clk}}}$$

Two important facts of this block are:

- It is important to saturate the slope. Otherwise, the massive injection of spikes will saturate the complete system.
- The validity of this block is limited by time. It fits with the time-limited connection between the premotor cortex and the primary motor cortex [32], but it is necessary to fine-tune the limit in order to reach the target. We use this time limitation to consider the GO signal as a disturbance for calculating the system stability.

### 4.4. Stability Analysis

**Figure 9.**
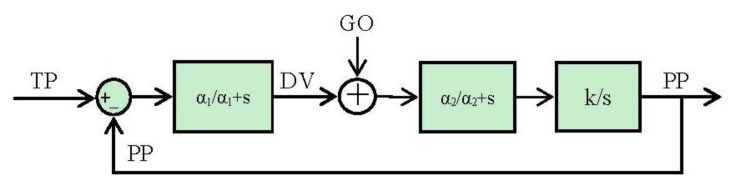
Block diagram for the VITE algorithm in the spike-based paradigm. We have included the low pass filter for the ramp multiplier.

It could be said that it is not necessary to study the stability of the system because it is open loop controlled, but the algorithm has an internal loop to generate the trajectory. In a biological way, this internal loop comes up from the corollary discharge signal as it has been explained. The starting-point is the block diagram resulted from mapping the VITE algorithm into the spike paradigm shown in Figure 9.

As the GO signal has a temporal life, it can be considered as a “caused” disturbance from the classical control theory point of view. According to this, the response of the system can be split in two parts, one due to the GO signal and the other one due to the target:

(8) $$\frac{PP_{GO}\left(s\right)}{GO}=\frac{\left(\overset{\alpha_{2}}{\;}/\underset{s+\alpha_{2}}{\;}\right)\left(\overset{k}{\;}/\underset{s}{\;}\right)}{1+\left(\overset{\alpha_{1}}{\;}/\underset{s+\alpha_{1}}{\;}\right)\left(\overset{\alpha_{2}}{\;}/\underset{s+\alpha_{2}}{\;}\right)\left(\overset{k}{\;}/\underset{s}{\;}\right)}$$

(9) $$\frac{PP_{TP}\left(s\right)}{TP}=\frac{\left(\overset{\alpha 1}{\;}/\underset{s+\alpha_{1}}{\;}\right)\left(\overset{\alpha_{2}}{\;}/\underset{s+\alpha_{2}}{\;}\right)\left(\overset{k}{\;}/\underset{s}{\;}\right)}{1+\left(\overset{\alpha_{1}}{\;}/\underset{s+\alpha_{1}}{\;}\right)\left(\overset{\alpha_{2}}{\;}/\underset{s+\alpha_{2}}{\;}\right)\left(\overset{k}{\;}/\underset{s}{\;}\right)}$$

Thus, the total response for the complete system is the addition of both [33]:

(10) $$PP\left(s\right)=PP_{GO}\left(s\right)+PP_{TP}(s)$$

The equivalence between the constants of the blocks in the block diagram and the parameters for the algorithm implementation is [21]:

(11) $$\omega_{\text{cut}-\text{off}}=\frac{f_{\text{CLK}}}{2^{N_{i}-1}\left(1+IG\_FD\right)}$$

(12) $$k=\frac{f_{\text{CLK}}}{2^{N_{k}-1}\left(1+IG\_FD\right)}$$

The parameters NBITS and IG_FD can be different for each block. However, IG_FD is computed as zero to avoid spike filtering [31]. Applying the Routh-Hurwitz stability criterion to the system, it is easy to find the following constraint:

(13) $$\frac{1}{2^{N_{1}-1}}+\frac{1}{2^{N_{2}-1}}>\frac{1}{2^{N_{K}-1}}$$

If we consider the same low pass filter (*N*_1_ = *N*_2_ = *N*), the constraint can be expressed as:

(14) $$N_{k}>N-1$$

The system suggests a trade-off between the value of *N_k_* and the response speed. The higher the value, the slower the speed achieved. Thus, the value of *N_k_* should assure the stability constraint and it also allows a quick response.

Once the stability of the system has been shown, some aspects must be mentioned. There is a big difference between the classical continuous signals and these spike-based systems: the idle state as we understand it (zero value for a signal) does not exist in a spike system [34]. In our system we have an element (Hold and Fire) where a spike comparison is performed with a temporal window to decide whether to fire a spike or not; the joint of this element with the other integrators and generators can cause a spurious minimum firing rate. For example, we could be considering an idle state if there were a firing rate of one spike per hour and it was not completely true. This fact can be understood as a white Gaussian noise. Nevertheless, to avoid this noise effect, the GO signal has a temporal life ensuring non added spikes to the reference. This temporal validity of the GO signal could also provoke a non-reaching if its effect is not enough. A tradeoff between the stability of the system and successful reaching is established.

## 5. From Simulations to Implementation: The Hardware

### 5.1. Introduction

**Figure 10.**
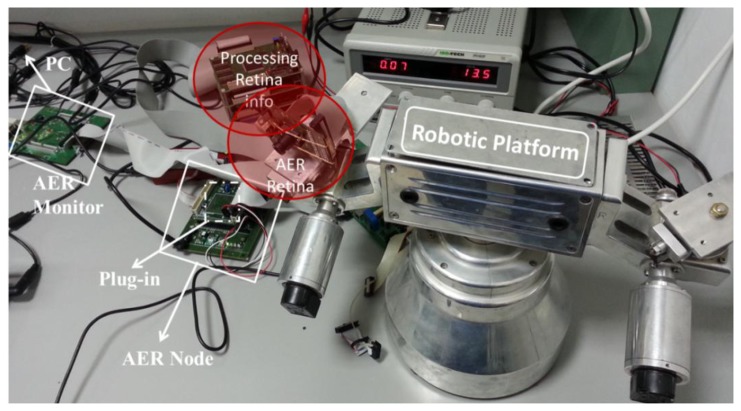
Setup designed to check the algorithm translation. Almost all the elements are shown. Behind the robotic platform is the actuation layer, where the spikes from the processing layer are received and transmitted to the motors.

In this section, we are going to use the VITE translation into spikes paradigm to move to several boards to check the algorithm behavior. The setup (Figure 10) consists of two separate parts: the visual perception and the robot actuation. Visual perception is composed of an AER Dynamic Vision Sensor (DVS) retina sensor and its spike-based processing elements for object detection and targeting. These elements are two AER processing layers working in cascade for firstly detecting different objects, and secondly tracking them even with crossing trajectories. Several objects can be detected and targeted in parallel. The maximum number of parallel objects to be processed depends on the FPGA available resources. For a Spartan II 200 FPGA from Xilinx up the maximum are four objects. The first layer starts “seeing” the complete visual field from the DVS for all the parallel object detectors. When an object is detected, the mass center is used to close the visual field to be observed in order to track only this detected object. The tracking process is done by a cascade spike-based processor and it offers also the speed. The output of the system offers not only the center position of the tracked object but also the speed in pixels per second. The system is fully hardware implemented on FPGA (Spartan II 200) [24].

**Figure 11.**
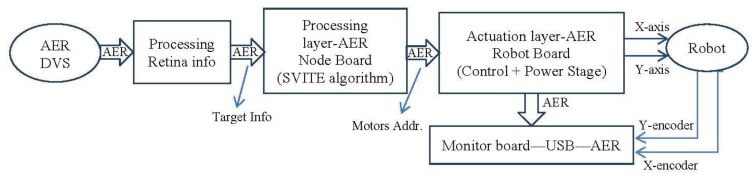
Model of the setup shown in Figure 10. The AER retina delivers the addresses of spiking pixels. Then, just the address of the tracking object's center is fed as the target position to the architecture; it is split for both algorithms which control each axis. The encoders are used just to provide information about the movement of each axis. All the arrows represent AER buses.

The robot actuation part consists of two FPGA boards and a robotic platform (Figure 11). The visual perception part delivers the target position in real time to the first FPGA board of the robot actuation part, which acts as the motor-cortex or central pattern generator unit, from an electronic point of view. Then, this board uses the AER protocol to communicate with the second FPGA, which is the second layer in the hierarchy: the actuation layer. Biologically, it is like the spinal cord or the brainstem. Finally, this second layer applies the commands to reach the target through the motors that mimic the biological muscles in the robotic platform. The following is structured with two subsections to describe the processing and actuation layer, respectively.

### 5.2. Processing Layer

This layer receives the reference from the target detected by the AER vision processing layer and applies the SVITE algorithm to produce the non-planned trajectory within the blocks exposed in previous sections.

It is implemented in the Spartan-6 Xilinx FPGA present in the AER-node board (developed under the VULCANO project). This board allows x4 2Gbps high speed bidirectional serial AER communications over Rocket IO GTX transceivers using SATA cables. AER-node provides several daughter boards for extending the functionality/connectivity. We have used a daughter board that provides a USB microcontroller that communicates with the FPGA using Serial Peripheral Interface (SPI). This USB interface has been used for configuring the spike-based blocks of the VITE algorithm directly from MATLAB (Further information about AER-node and other PCB design of the RTC lab of the University of Seville can be found at http://www.rtc.us.es/). The visual processing layer delivers a reference to this processing layer. The form of this reference is an address (in AER format) that matches with the pixel which is firing at the center of the target object. These addresses are usually decomposed in (x, y) coordinates of the retina visual reference. By means of experimental findings, generator resolution is:

(15) $$\text{Resolution}=65\times 2^{10}/\overset{2^{\text{NBITS}-1}}{\;}(\frac{\text{degrees}}{\text{spikes generator step}})$$

where the parameter NBITS is the number of bits selected to implement the spike generator that supplies the target. With this data, we can translate the target information from the retina to a suitable reference for each algorithm.

The algorithm is split into as many parts as motors there are in the robotic platform. In this work, the robotic platform has four degrees of freedom: two axes with two motors per axis. We use two SVITE: one for the x-axis and another one for the y-axis. Each SVITE output is sent to two motors (same behavior). Furthermore, this division is in agreement with the pursued goal of producing an intended but synchronous movement. If we have two algorithms, it is possible to adjust each GO signal to succeed.

One problem derived from the replication of the algorithm is related to the spike production. All the algorithms fire nearly at the same time and although AER handshake protocol takes a short period of time to communicate with the bus, a few spikes could be lost. Indeed, the access to the bus can crash with the spike production of the algorithms, leading to spike loss. Thus, to avoid this loss two options are available:

Include a First-In-First-Out (FIFO) buffer memory that is used to palliate sporadic high speed problems in the AER communication at the output of each algorithm; the arbiter will access the memories to carry out the communication.

Avoid the use of an arbiter and communicate the instances by using just a pair of wires to transmit the spikes (point to point communications from processing to actuation FPGAs).

In the presented architecture, both schemes have been studied: communication with the next layer by AER protocol (only one pair of addresses is needed; one per each algorithm) and with a pair of wires are considered. The differences are shown in the results section.

### 5.3. Actuation Layer

This layer will adapt the information received in order to feed the motors of the robotic platform. It receives addresses of both algorithms and produces the spikes for the motors.

It is implemented in the Spartan-3 Xilinx FPGA present in the AER-Robot board [21] (developed under the SAMANTA-II project (Multi-chip AER vision system for robotic platform II (SAMANTA-II), October 2006 to September 2009), which is able to drive DC motors through opto-coupler isolators and full L298N bridges for motor actuation.

The robotic platform is a stereo-vision robot with four degrees of freedom powered by DC motors. Although, the motors have an isolated movement, at this moment, they are coupled in pairs, one for each axis according to the usage of just one retina. Thus, each axis is fed with one algorithm, so we have one algorithm for the pair of motors of the axis. The power supply requirement of the motors is 24 Vdc. The manufacturer of the motors is Harmonic Drive and the model is RH-8D6006. The structure of the robotic platform is made so that the motors of the y-axis are crossed to their axis and have a transmission belt to move the arm. With regard to this structure, we fed the y-axis motors with the position (because it is needed to hold the spike transmission) and the x-axis motors with the speed profile.

We propose to use PFM to run the motors because it is intrinsically a spike-based solution almost identical to the solution that animals and humans use in their nervous systems for controlling the muscles. Nevertheless, we need to adapt the spikes because the digital clock of the boards is fixed at 50 MHz resulting in a spike width of 20 ns and this signal is very fast and the spikes too short for the motors model of the robotic platform [21].

To compute the maximum and minimum spiking rate allowed we must look in detail at the board components and the DC motor, respectively. On the one hand, for maximum firing rate, the power stage of the board consists of an optical isolator and H-bridges, as we have mentioned. Both components have some features regarding the switching frequency: for the H-bridge it is fixed at 40 KHz, but it is recommended to work at 25 KHz (minimum period of 40 μs) to avoid malfunctioning. As for the isolators, there is no maximum switching frequency defined, but two important temporal restrictions must be considered: 6 μs and 5 μs for raise and fall, respectively.

Merging these data, it results into a maximum firing rate of 25 KHz (40 μs of minimum period), and within this max rate the spike width can be solved. Using the minimum period of 40 μs and taking into account the temporal restrictions of the isolators, it results in a time period of 29 μs as maximum width. We have chosen a secure width of 25 μs for margin and to spread out the spikes up to 750 clock cycles. Definitely, with these data, the maximum switching frequency will be 25 KHz and the spike width 750 clock cycles.

On the other hand, to compute the minimum spiking rate allowed it is necessary to analyze the target actuator (DC motor in our case). A DC motor acts as a low pass filter and the transfer function can be calculated using the parameters from the manufacturer [33]. This function and, particularly, its step response allow us to select the motor's minimum switching frequency (maximum period) suitable to follow an input properly. The step response calculated illustrates an approximate total time of 40 ms to follow the input. Therefore, we are going to select a lower order value with a little margin: 1 ms of maximum period, so a minimum frequency of 1 KHz for the incoming spikes.

These two limits will allow us to build up the empirical table that maps the vision reference system and the movement produced at the platform. To sum up, we have the operating margin for the motors: from 1 KHz to 25 KHz and the spike width as 750 clock cycles. Notice that, if we make the spike injection in the GO block lower than ten percent of slope, it would not cause any movement at all because the motor will filter the spikes. In contrast, a much higher slope could saturate the system without a closed loop control.

### 5.4. Hardware Resources Consumption

In general, to measure the hardware consumption in a FPGA, two points should be considered: the dedicated resources included to build up complex devices such as multipliers and the configurable logic blocks (CLBs) for general purpose. The algorithm does not use any complex structure. It just needs counters and simple arithmetic operation resources. Therefore the measurements are focused into the available slices at the FPGA.

We have synthesized the algorithm, including a spikes rate coded generator [35], a spikes monitor [36] and the interface with other neuromorphic chips for a correct debugging and a useful integration. Table 1 presents the data for the device with the report obtained.

In this table, the first column describes the element implemented for each case. The next column shows the amount of slices needed to synthesize the units at the FPGA. The following column represents the maximum number of units that could be allocated inside the FPGA. Finally, in the last column the total capacity of the device for all the synthesis performed is shown.

The results evidenced a low hardware resource usage when an isolated algorithm is implemented, just one per cent. Also, it is remarkable that the interface with other neuromorphic chips almost does not provoke an increment in the hardware resources consumption (only four slices). Consequently the final implementation for a complete architecture will consist of the algorithm and the interface. However, the design and test phases need the monitor in order to check the right behavior of the algorithm.

All the results presented in this section correspond just to slice consumption. The FIFO included within implementation uses dedicated memory blocks already present in the device, so it is not computed.

If we compare the maximum number of algorithms that can be allocated at the FPGA (that corresponds to the degrees of freedom (DoF) controllable in our architecture) with the iCub Robot necessity [18], it shows a great advantage using our approach. We can control up to 95 DoF (without monitor) in comparison with iCub platform which allows 53 DoF.

### 5.5. Power Consumption

The power consumption of the design implemented can be divided in three different parts: the device static, design static and design dynamic power consumption. The device static power consumption is also called the off-chip power and it is referred to the power consumption of the board without any configuration. The design static power consumption is the power used when the design is just programmed into the board but it is not running. Finally, the dynamic power consumption is referred to the power used by the design when it is running.

We have used the XPower estimator tool from Xilinx to get the device static and design dynamic power consumption. The results are: 0.113 W for the device static power and 0.027 W for the design static power. The design dynamic power is obtained by computing the difference between the real measurement, when the algorithm is running, and the addition of device static and design static power consumption. The power consumption measured is 3.4 W, thus the design dynamic power is 3.26 W.

## 6. Results

This section presents several results for the whole design. These results aim to show the evolution from the original VITE algorithm design by Grossberg [11], going through its translation into spikes (SVITE), to a real robotic platform. Then, the two options explained for the communication between the actuation and processing layer are illustrated to check the translation done. Finally, we want to show the performance of the designed control system. Afterwards, we present some discussions.

**Figure 12.**
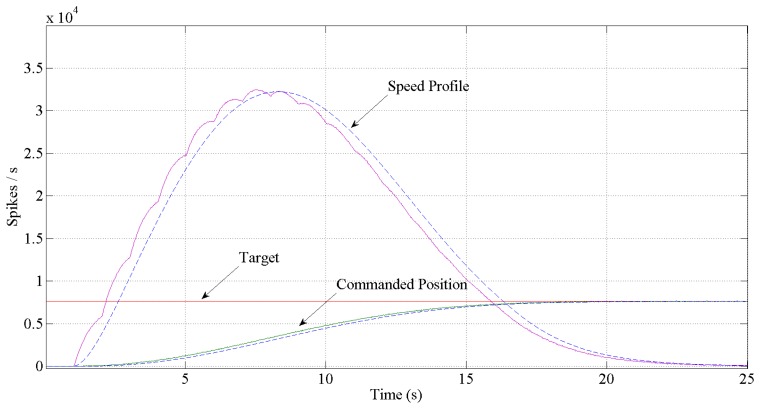
Performance achieved corresponding to one percentage slope in GO signal. Dotted lines are simulated in front of measurement solid lines. The bell shape profile signals represent the speed. The ripple in the spike-base behavior is due to the function that transforms the spikes into a continuous signal. The target is the same for both simulated and measurements signals and it is represented as a firing rate. It takes a total of 17 s to reach the target if we look through the position.

**Figure 13.**
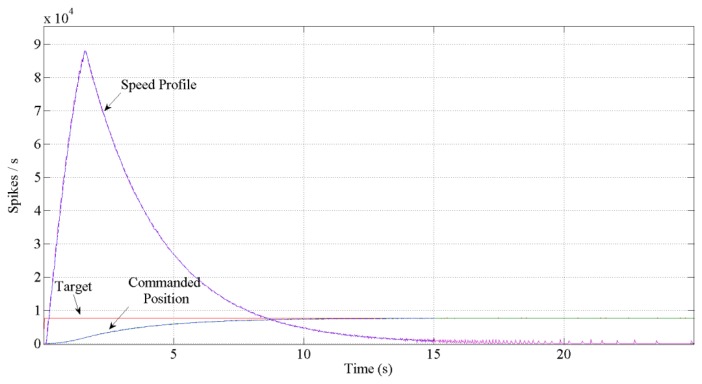
Performance achieved corresponding to ten percentage slope in GO signal. Dotted lines are simulated in front of measurement solid lines. The bell shape profile signals represent the speed. The ripple in the spike-base behavior is due to the function that transforms the spikes into a continuous signal. The target is the same for both simulated and measurements signals and it is represented as a firing rate. It takes a total of 12 s to reach the target if we look through the position.

**Figure 14.**
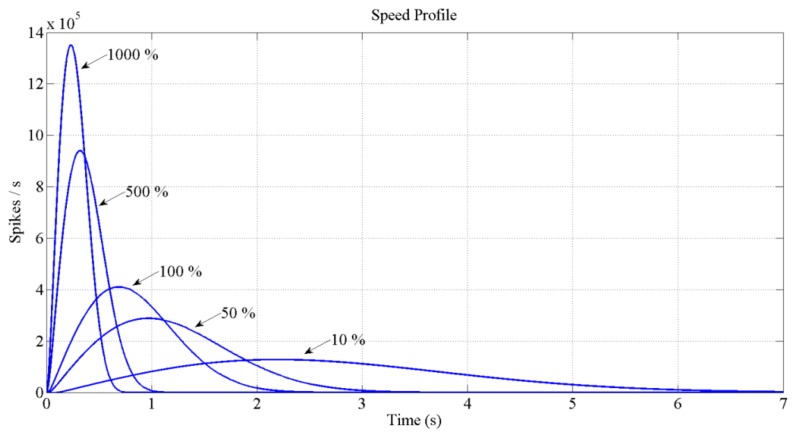
Speed profiles achieved by modifying slope_counter parameter of GO block. Making a comparative between slow and fast movements we can appreciate that the peak velocity is reached later for faster movements if entire length is considered.

**Figure 15.**
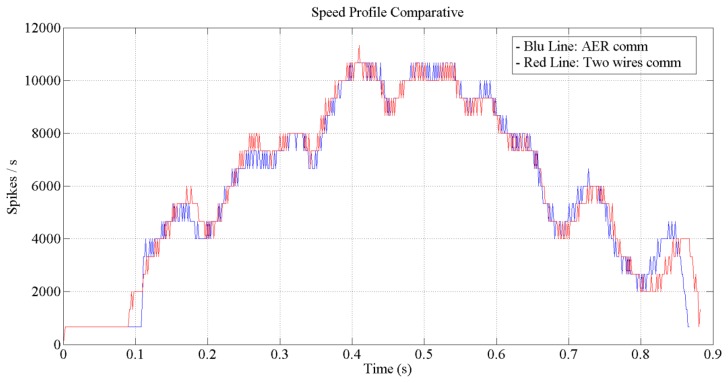
Speed profiles read out from motor encoders. The blue profile is read with an AER communication and the red profile is from a pair of wires communication. The input was an AER address matching coordinate 125 for the x-axis.

**Figure 16.**
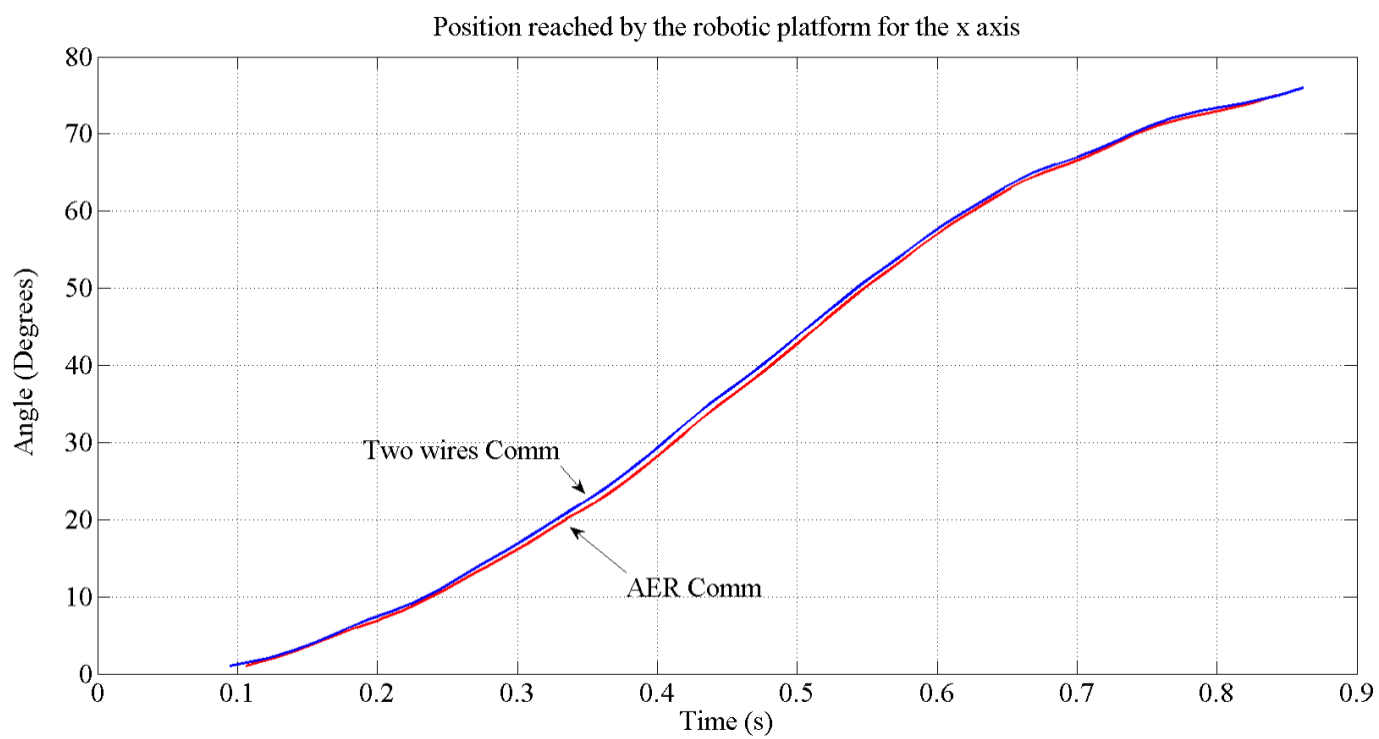
Position reached by the motors for the x-axis when two different communication strategies are used.

**Figure 17.**
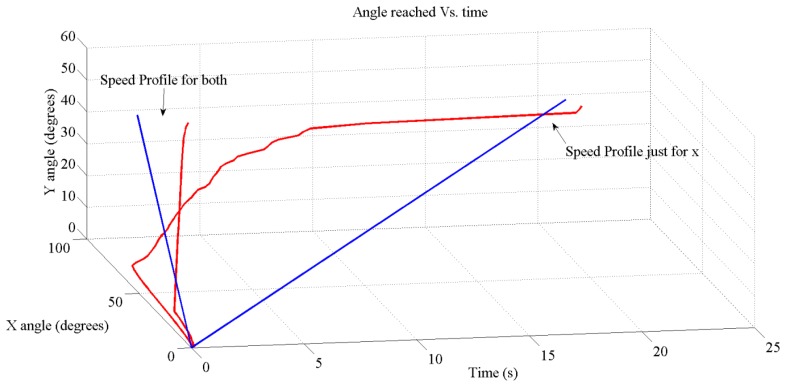
Angle *vs.* time reached for both axis with (125, 90) input. The retina has 128 × 128 pixels. The communication between neural boards was made by AER. The red lines show the trajectory followed by the robot when we used the speed profile for both axis and just for the x-axis; the blue lines represent the motion delivered by the DVS sensor.

**Figure 18.**
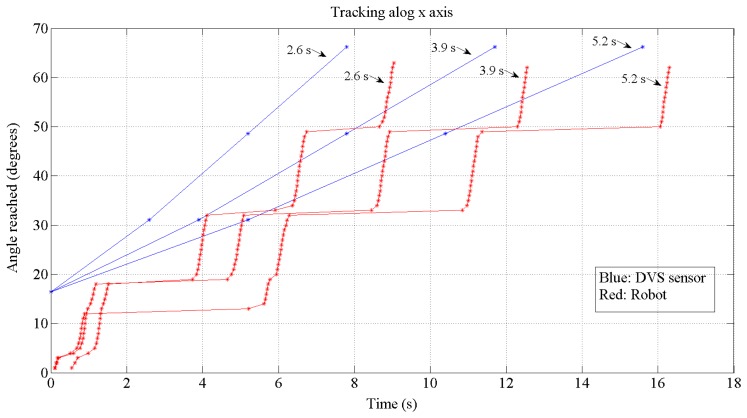
Angle *vs.* time tracked for the x-axis. The input is a go and return along the x-axis in the frame of reference of the retina. The red points show the angle trajectory followed by the robot and the blue points show the targets delivered to the robot.

These results have been achieved by means of these tools: on the one hand, with MATLAB and Xilinx System Generator we have managed the theoretical and simulation scenarios to get the simulated results (part of the Figures 10, 11, 12, 13 and 14); on the other hand, the software suite from Xilinx was used to synthesize into the FPGA devices of the boards at the hardware setup to get the running results (Figures 15, 16, 17 and 18).

Figures 12 and 13 show the expected behavior of the algorithm translated into spikes paradigm against the behavior of the original design of the algorithm by Grossberg. The dotted lines are taken from simulations of the original VITE algorithm; solid lines show the same data but measured in the boards with a special AER monitor [36,37]. The speed profile is taken before the integer block (the Integrate and Generate block in Figure 5) and the position orders at the output. The accuracy for both signals is highly precise and it suggests the opportunity of succeeding with a fully spike-based robot controller. Figure 14 shows simulation data for the speed profile achievable when the parameter slope_counter in GO block goes through different values causing 10, 50, 100, 500 and 1000 percentage slopes. The bell shape profiles confirm the studies in [27] where it is said that as faster is the movement the higher asymmetric speed profiles are performed. At the description of the layers we presented two options for the communication between both: AER communication or just a pair of wires carrying the spikes. In Figure 15, both options are shown regarding speed profile. This graph does not reveal any change at the firing rate when an AER communication is applied between the layers. This fact allows us to say that AER communication is a good strategy to connect spike-based processing and actuation without any modification in the spiking rate transmissions. These two tests concern only the x-axis which is the one fed by the speed profile. The slope used was 10%.

In Figure 16, the position (angle) reached for each type of communication is shown. The reference ordered for this movement was 75 degrees. As we can see, with both types of transmission, the target will be reached by the platform. The blue line represents the position reached when a two wires communication is performed and the red line represents the position reached when an AER communication is used. The tiny difference between both communication modes are due to the fact that within AER, a handshake took place to access the bus. The test also reveals a maximum speed of 83 degrees per second on the x-axis.

Despite using or not an AER communication or two wires, one of the more attractive items of the algorithm was that it is possible to generate synchronous movements by controlling the GO signal independently for each motor. Figure 17 shows the real measurements of the position reached when the target is fixed at (125, 90) in the frame of reference of the retina. This becomes an angle of 75 degrees for the x-axis and 48 degrees for the y-axis in the frame of reference of the robotic platform. Moreover, the figure shows the translation of the target delivered by the sensor.

Since our robotic platform has a special architecture that leads us to use the position commands for the y-axis and the speed commands for the x-axis (to hold the target position at the end) it is not easy to produce synchronous movements; indeed it is impossible because the position commands are slower than the speed commands. Nevertheless, we have fed the y-axis also with the speed profile (although it does not hold the position at the end) to check how synchronized a movement can be done with this algorithm; the result is extremely accurate if we compare it to the target provide representation.

Turning to the slower trajectory and looking at it, the x-axis gets the position commanded in approximately one second and then starts the movement in the y-axis. If we compare the theoretical signal delivered to the motors (Figure 13) and the movement achieved (reads out from the encoders of the motors and using jAERsoftware tool [38]) shown in Figure 17, it reveals a couple of comments regarding time reaching length. For the x-axis, the one commanded by the speed profile, it is quite different, but in both cases, they have the same maximum firing rate and for the y-axis, commanded by the position, we found nearly the same length. The reason for the difference at time length in the x-axis can be understood with these two points: non-feedback used, which means once the motor starts running we do not have any inertia control, non-feedback from any sensor.

However, the accurate movement achieved when both axis use the speed profile leads us to think about getting gestures if all the motors were able to be fed with the suitable velocity profile to hold the position.

The test performed in Figure 16 to check the communication strategy revealed a time to reach the top movement of 0.9 s (the test was done with the maximum movement achievable by the robot: from 0 to 127 coordinate of the retinas' frame). If we consider this reaching time and joint it with the latency shown in Equation (7), it results in a minimum of 1.1 s distance between switching the target. It will be the limit to the robot to be able to follow in the right way.

Thus, in Figure 18 we have performed a real test to check the tracking properties of the robot. The test is done just for the x-axis which is the one commanded by the speed profile. The angle fed by the retina is calculated using jAER. For these tests, the target has been delivered to the processing layer by DVS sensor with three difference time distances: 2.6, 3.9 and 5.2 s for each one. It is possible to detect the latency of 0.1 s at the beginning. The processing time can also be calculated by computing the time between the target delivery and the start of the motion minus the fixed latency. It results 0.5 s.

The difference between the angle reached by the robot and the motion represented is due to the resolution obtained by the encoders of the motors and also to the cumulative error in an open-loop controller within many targets without calibration between them. Finally, empirical tests reveal an accurate tracking by the robot when the distance between target deliveries is at least 2 s.

Finally, to conclude the results section, here are some important considerations revealed by them:

- Lower spiking rates: we have higher spiking rates at the simulations than in real results; for real robotic platforms, the firing spiking rate should be adapted to the motors in order to succeed with the PFM control. Also, the rates are limited by the electronic components of the used boards.
- Life time limited: the signals read out from the encoders are shorter than they were supposed to be taking simulation results. This fits with the non-feedback used from the proprioceptive sensors; no motor inertia control at all.
- Delay support: the same as the primitive algorithm supports delay [39], our translated algorithm could also support them: if the GO signal is shot after or before the target is submitted, it will only cause a delay or a jerk due to the higher starting speed, respectively.
- We have to choose an AER or a couple of wires communication. If the robotic platform has many DoF, the AER protocol may be used. Otherwise, a pair of wires communication has an accurate enough behavior up to eight degrees of freedom (16 bits of AER bus).
- The mapping function was created with empirical results. Thus, the table has an intrinsic limitation: it was designed with specific parameters and it would cause the robotic platform to malfunction if we change them. Therefore, to check a right synchronized movement as VITE described it is needed to close the loop using proprioception sensors. Also, to make the algorithm portable to any robotic platform, this mapping function should not be constructed with empirical results.

## 7. Discussion: Connection between SVITE and Biological Movement

Although the physiological evidence to match theVITE algorithm with neurological behavior is clearly defined at the literature [40], in this section a connection between the SVITE and the biological movement will be established as long as we have added additional blocks and modified nearly all of them.

The biological movement has two different sources of sensory information: one called proprioceptive information or sensing coming from joint position and muscle tension sensors and another flow regarding target situation given by the retina.

The information given by the retina goes into the algorithm and starts the Difference Vector (DV) computation; this previous computation matches with activity registered in the premotor cortex before the movement begins in biological motion by specific neuron cells [41]. Furthermore, the GO signal appears between the premotor cortex and the primary motor cortex. This signal is the shooting for the movement. Also, the DV signal is not delivered straight to motors. There are three spike-based blocks in between. This matches the concept of “project to interneurons” rather than “directly to motor-neurons” [32].

As we have seen up to this point, the movement is generated directly from the algorithm's output or directly from the brain's output in a biological way. We suggest a feedforward model, without either a proprioceptive sensor nor information from the retina at this step of the processing because they will be included in the next step: the second phase FLETE which includes feed-back processing.

In general terms, the feedback allows a precise reaching movement and also updating the present position during a passive movement. In the present work it is assumed that the robot reaches the position commanded. This feed-forward model does not accept delays, noise and the motor commands must be highly precise [32].

The next step (FLETE) shall include several feed-back loops: one short local loop at the muscle (mimicked by the motors in a robot) as an automatic gain control, a second short local loop provided by the retina in the visual sensory area and at last a large loop from proprioceptive sensors.

## 8. Conclusions

A fully neuroinspired architecture has been presented: from an AER retina to PFM controlled motors. The system aims to keep as many features in mind as possible from the biological intended movements:

- Hierarchy system with a processing and an actuation layer like the brain and brainstem.
- Some activity previous to the movement (the latency and computing of difference vector) like the previous activity detected at the premotor cortex.
- GO signal is in the edge of the premotor and primary motor cortex and its hardware implementation based on spikes saves computational costs because it is done as an addition instead of a signals multiplication.

This research meets its goals with neuromorphic engineering ones, that is: mimicking the neuronal system behavior in order to develop useful applications. The controller designed uses only 2% of the Spartan 6 FPGA and the power consumption is 3.4 Watts excluding the motors. The results reveal the accurate use of AER protocol for actuation purposes. The controller can be replicated up to one hundred times to control complex robotic structures with such DoF.

The open-loop neuro-controller designed and implemented is able to reach, in a synchronized way, any position commanded by the output of an accuracy tracking done by a cascade architecture based on DVS sensor.

Further research will provide a full feedback architecture including passive movement updating and proprioceptive information from muscles for fine tuning. With this advances we aim to use the system to improve accuracy in real-time running robots.

## Figures and Tables

**Table 1.** Hardware resources consumption by the Spartan 6 1500 device.

	**Number of Slices**	**Max. Blocks in** **the Device**	**Use by** **One Block**
Algorithm	238	96	1.033%
Algorithm plus monitor	533	43	2.31%
Algorithm plus interface	242	95	1.05%
Algorithm plus monitor and interface	537	42	2.33%
